# Digital Guardian Angel Supported by an Artificial Intelligence System to Improve Quality of Life, Well-being, and Health Outcomes of Patients With Cancer (ONCORELIEF): Protocol for a Single Arm Prospective Multicenter Pilot Study

**DOI:** 10.2196/45475

**Published:** 2023-04-21

**Authors:** Joaquim Reis, Luzia Travado, Alexander Scherrer, Thanos Kosmidis, Stefanos Venios, Paris Emmanouil Laras, Gabrielle Oestreicher, Markus Moehler, Margherita Parolini, Alessandro Passardi, Elena Meggiolaro, Giovanni Martinelli, Elisabetta Petracci, Chiara Zingaretti, Sotiris Diamantopoulos, Maria Plakia, Charalampos Vassiliou, Suheib Mousa, Robert Zifrid, Francesco Giulio Sullo, Chiara Gallio

**Affiliations:** 1 Institute of Biophysics and Biomedical Engineering Faculty of Sciences University of Lisbon Lisboa Portugal; 2 Institute for Industrial Mathematics Fraunhofer-Institut für Techno- und Wirtschaftsmathematik (ITWM) Kaiserslautern Germany; 3 Careacross London United Kingdom; 4 Suite5, Data Intelligence Solutions Limited Limassol Cyprus; 5 Maggioli SPA Santarcangelo di Romagna Italy; 6 Universitaetsmedizin der Johannes Gutenberg-Universitaet Mainz Mainz Germany; 7 IRCCS Istituto Romagnolo per lo Studio dei Tumori “Dino Amadori”, IRST S.r.L. Meldola Italy; 8 Exus Software London United Kingdom; 9 Innosystems Athens Greece; 10 MCS Datalabs Berlin Germany

**Keywords:** eHealth, artificial intelligence, quality of life and well-being, supportive cancer care, mobile phone, cancer support, artificial intelligence–based recommendations

## Abstract

**Background:**

According to Europe’s Beating Cancer Plan, the number of cancer survivors is growing every year and is now estimated at over 12 million in Europe. A main objective of the European Commission is to ensure that cancer survivors can enjoy a high quality of life, underlining the role of digital technology and eHealth apps and tools to achieve this.

**Objective:**

The main objective of this study is the development of a user-centered artificial intelligence system to facilitate the input and integration of patient-related biopsychosocial data to improve posttreatment quality of life, well-being, and health outcomes and examine the feasibility of this digitally assisted workflow in a real-life setting in patients with colorectal cancer and acute myeloid leukemia.

**Methods:**

A total of 60 patients with colorectal cancer and 30 patients with acute myeloid leukemia will be recruited from 2 clinical centers: Universitätsmedizin der Johannes Gutenberg-Universität Mainz (Mainz, Germany) and IRCCS Istituto Romagnolo per lo Studio dei Tumori “Dino Amadori” (IRST, Italy). Psychosocial data (eg, emotional distress, fatigue, quality of life, subjective well-being, sleep problems, and appetite loss) will be collected by questionnaires via a smartphone app, and physiological data (eg, heart rate, skin temperature, and movement through step count) will be collected by a customizable smart wrist-worn sensor device. Each patient will be assessed every 2 weeks over their 3-month participation in the ONCORELIEF study. Inclusion criteria include patients with the diagnosis of acute myeloid leukemia or colorectal cancer, adult patients aged 18 years and older, life expectancy greater than 12 months, Eastern Cooperative Oncology Group performance status ≤2, and patients who have a smartphone and agree to use it for the purpose of the study. Exclusion criteria include patients with a reduced cognitive function (such as dementia) or technological illiteracy and other known active malignant neoplastic diseases (patients with a medical history of treated neoplastic disease are included).

**Results:**

The pilot study started on September 1, 2022. As of January 2023, we enrolled 33 patients with colorectal cancer and 7 patients with acute myeloid leukemia. As of January 2023, we have not yet started the data analysis. We expect to get all data in June 2023 and expect the results to be published in the second semester of 2023.

**Conclusions:**

Web-based and mobile apps use methods from mathematical decision support and artificial intelligence through a closed-loop workflow that connects health professionals and patients. The ONCORELIEF system has the potential of continuously identifying, collecting, and processing data from diverse patient dimensions to offer health care recommendations, support patients with cancer to address their unmet needs, and optimize survivorship care.

**Trial Registration:**

German Clinical Trials Register (DRKS) 00027808; https://drks.de/search/en/trial/DRKS00027808

**International Registered Report Identifier (IRRID):**

DERR1-10.2196/45475

## Introduction

### Background

Cancer is the second leading cause of death worldwide, and 10 million deaths in 2020 were attributed to cancer [1]. In 2020, a total of 2.7 million people in the European Union were diagnosed with cancer, and another 1.3 million people lost their lives to it, including over 2000 young people [2].

According to the Europe’s Beating Cancer Plan, the number of cancer survivors is growing every year and is now estimated at over 12 million in Europe [3]. The Europe’s Beating Cancer Plan aims to ensure that patients with cancer, survivors of cancer, their families, and caregivers can enjoy a high quality of life (QoL), highlighting the role of digital technology and eHealth app and tools to achieve this objective. In the last decade, there has been an increasing use of eHealth (ie, the broad use of health information and communication technologies and networks to enhance patient-centered care delivery) in the delivery of patient-centered cancer care [4,5]. Among the tools that have been used are smartphone apps, wearable devices, SMS text messaging, and internet-delivered interventions [5]. However, there has been little use of artificial intelligence (AI) in psycho-oncology research.

ONCORELIEF is a European project funded by EU Horizon 2020 (grant 875392) whose main objective is the development of a user-centered AI system to facilitate the input and integration of patient-related biopsychosocial data to improve posttreatment QoL, well-being, and health outcomes. Measurable parameters include patient-reported outcomes about their unmet needs (eg, emotional distress, fatigue, sexual problems, and sleep quality), physiological data monitored by a wearable device (eg, heart rate and skin temperature), and demographic and health status data from medical records. This objective is achieved by establishing a workflow that connects health professionals and patients by means of assisting digital solutions.

### Concept and Study Architecture

**Figure 1:**
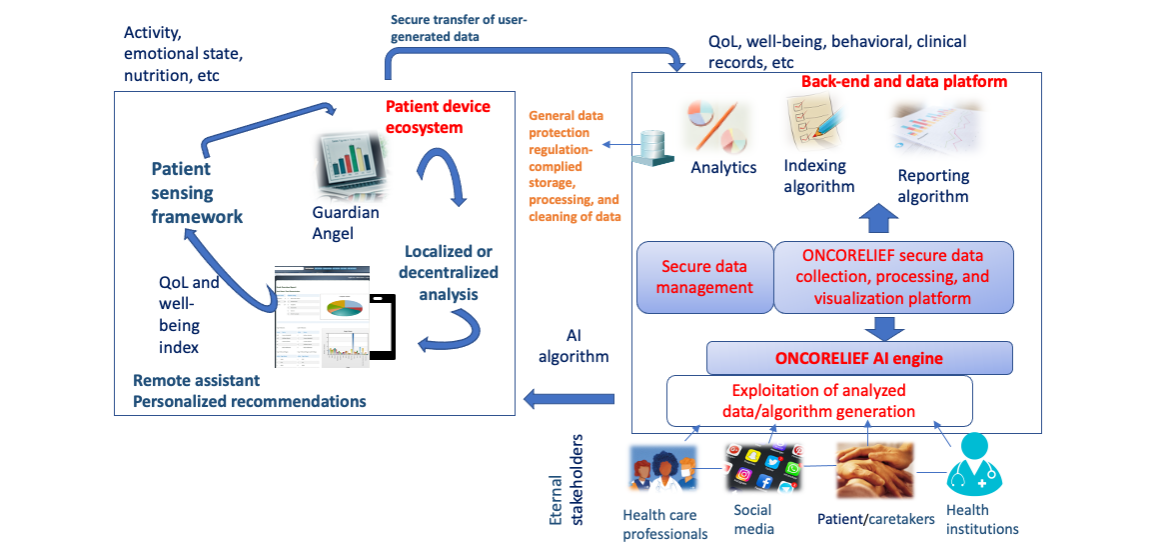
ONCORELIEF concept and architecture. The 5 main technological components of ONCORELIEF are the Guardian Angel—this is an app, compatible with portable devices operating with iPhone operating system and Android built upon the AI models generated by the ONCORELIEF platform; the back-end and data platform—this is where all the data are collected, processed, and used to deliver the services of the ONCORELIEF platform; the Big Data and AI engine—this resides on top of the back-end platform and exploits the insight derived from the analyses of the data as well as it is responsible for the interaction with the users of the Guardian Angel; the QoL and well-being index—this is used to monitor the QoL and well-being status and progress of the different users; and the sensing framework—this is the unobtrusive and pervasive, wearable, and embedded sensing ecosystem of the patient for data collection. AI: artificial intelligence; QoL: quality of life.

The way that the connection between health professionals and patients is done is presented in Figure 1, which illustrates the concept and architecture of ONCORELIEF with its 5 technological components: the Guardian Angel app, the back-end and data platform, the Big Data and AI engine, the QoL and well-being index, and the sensing framework.

### Novelty of the Study

**Figure 2:**
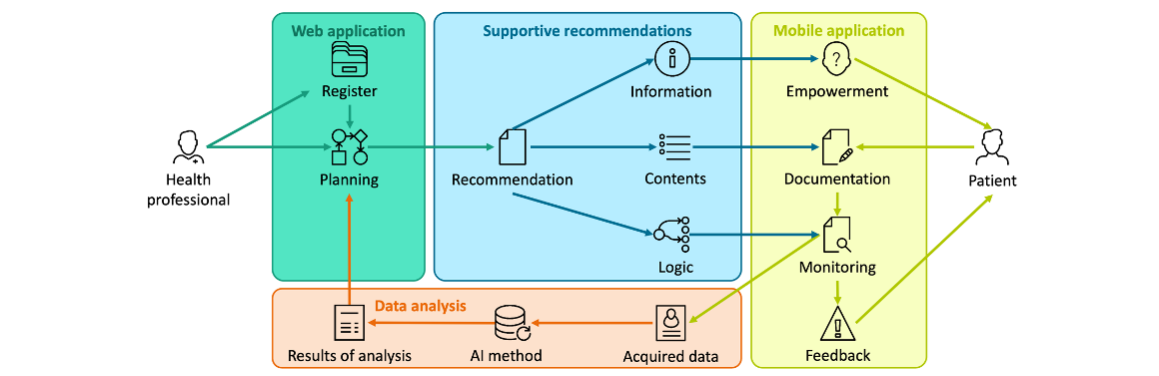
General AI solution approach with the core components and main information flows. The thick arrows indicate the main process steps of the approach. (Icons: Line icons [iconsmind.com] and Windows 8 icons [icons8.com]). AI: artificial intelligence.

A main innovation of ONCORELIEF is that all data will run on a digitally assisted workflow of posttreatment supportive patient care supported by an AI system (see Figure 2) [6]. This workflow comprises the planning of supportive recommendations with a web-based app by the health professional, the documentation and monitoring of recommendations with a mobile app by the patient, and analyzing the AI-based data of the achieved progress as preparation for the next planning step.

The specific realization of this approach depends on the circumstances of the application scenario, such as the preferred level of detail of the recommendations, the information contents of the acquired data, and the amount of reference data available for the training of AI methods. The realization would feature a provision of suitable supportive recommendations by the register, adequate concepts for their documentation and monitoring, and an appropriate selection of AI methods for data analysis. In this sense, the ONCORELIEF pilot study features text-based informative recommendations, the documentation of recommendation outcomes with standardized health questionnaires and vital parameter data measured with wearable sensor devices, and a data analysis with methods from statistics and statistical learning such as automated classification and artificial neural networks, respectively.

The ONCORELIEF pilot study is designed with the aim to examine the feasibility of this digitally assisted workflow in a real-life setting on 2 quite different types of cancer: colorectal cancer and acute myeloid leukemia. These represent cancers with different biological characteristics, different treatment pathways, and specific patient needs [7-10]. In this paper, we describe the study protocol of a multicenter pilot study. There are four key questions to be examined: (1) It is feasible to assess the unmet needs of patients with cancer, after-treatment, through questionnaires included in a smartphone app (ie, Guardian Angel)? (2) It is feasible to collect physiological (eg, heart rate) and behavioral (eg, movement through step count) data from a wearable smart device connected with the app? (3) It is feasible to generate, with the support of AI, a composite and ongoing QoL and well-being Index that indicates continuously the balance of met and unmet needs of patients with cancer? (4) It is feasible to deliver timely recommendations for patients based on patients’ data inputted from the Guardian Angel app and a wearable device?

## Methods

### Study Design

**Figure 3:**
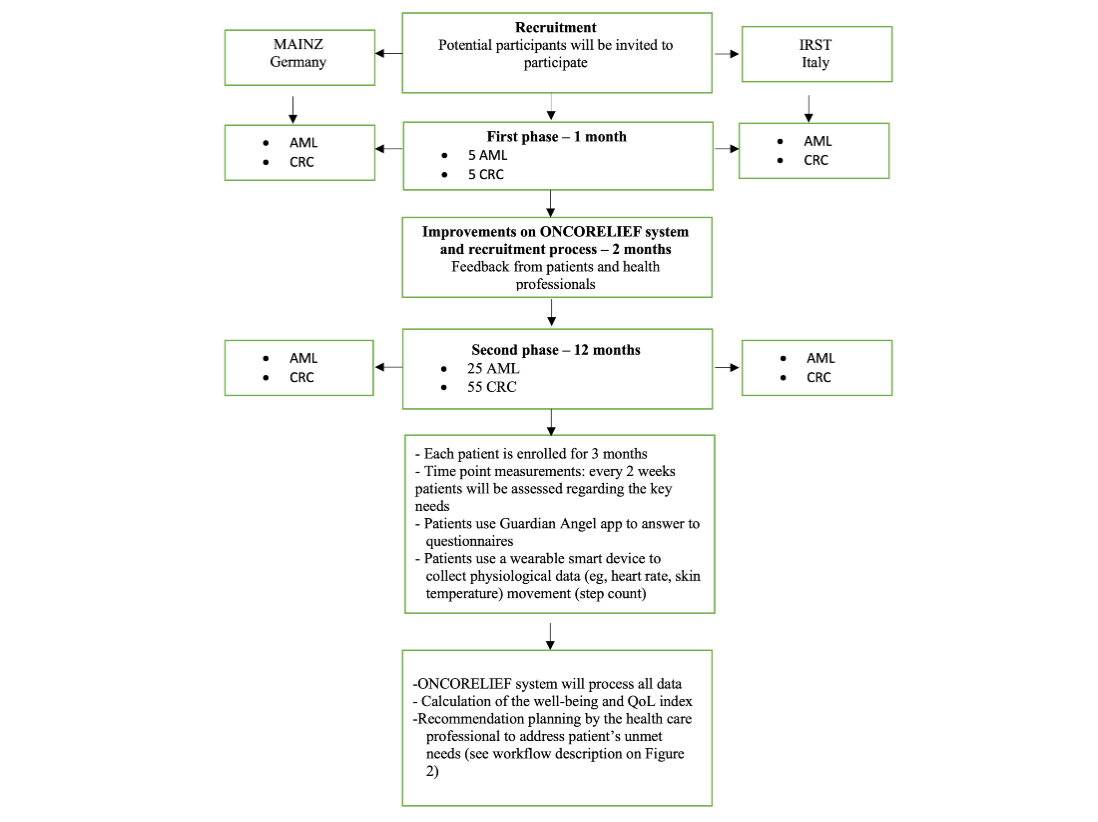
The flow of patients throughout the study. AML: acute myeloid leukemia; CRC: colorectal cancer; IRST: Istituto Romagnolo per lo Studio dei Tumori; QoL: quality of life.

This 2-group pilot study is aimed to evaluate the feasibility of the ONCORELIEF system in real-life clinical settings for patients with colorectal cancer and acute myeloid leukemia. It will run in two phases: (1) a prepilot phase is a 1-month training of the entire system. A total of 10 patients (5 with acute myeloid leukemia and 5 with colorectal cancer) will be enrolled and provided with the ONCORELIEF digital tools (ie, the Guardian Angel app and the wearable device). Based on the feedback of patients and health professionals, improvements will be made to the entire system over a period of 2 months. (2) The second phase will last 12 months (see Figure 3).

### Clinical Centers

The study will be conducted in 2 clinical centers: Universitätsmedizin der Johannes Gutenberg-Universität Mainz (Mainz, Germany) and IRCCS Istituto Romagnolo per lo Studio dei Tumori “Dino Amadori” (IRST, Italy).

### Study Participants

Inclusion criteria include (1) patients with the diagnosis of acute myeloid leukemia or colorectal cancer, (2) adult patients aged 18 years and older, (3) life expectancy greater than 12 months, (4) Eastern Cooperative Oncology Group performance status [11] ≤2, and (5) patients who have a smartphone and agree to use it for the purpose of the study.

Exclusion criteria include (1) patients with a reduced cognitive function (such as dementia) or technological illiteracy and (2) other known active malignant neoplastic diseases (patients with a medical history of treated neoplastic disease are included).

### Recruitment and Enrollment

Figure 3 describes the flow of patients throughout the study. All patients will be treated and monitored for their disease according to the local clinical practice, and no changes in treatment or follow-up will be decided based on participation in this study. At IRST Italy, patients in follow-up at clinical sites for acute myeloid leukemia or colorectal cancer disease and those considered eligible by the study oncologists will be invited to participate in this research. The oncologist will inform patients of the aims and procedures of the study. At Mainz, patients are prescreened by the treating health care professional (HCP) for eligibility and are introduced to the study by the HCP during their regular clinic visits. If the patient is interested, the HCP will give the patient’s name and the time of the next clinic visit to the study coordinator or study nurse. At the patient’s next visit, the study coordinator will meet the patient and enroll the patient by following the enrollment procedures.

All patients will be informed that the participation is voluntary and that they are allowed to interrupt their participation whenever they want and for any reason. This will not prejudice the patient’s subsequent care. Patients will also be informed about relevant data protection and privacy legislation. A written, signed, informed consent form from the patient must be obtained before study entry. After signing informed consent, patients will be registered into ONCORELIEF electronic data capture system. Only deidentified data will be collected, including the data collected by the app or wearable device. Each patient will be assigned a unique alphanumerical ID, and only clinical site staff will have access to patients’ personal data and reidentification key. The anticipated total number of recruited patients will be 30 acute myeloid leukemia and 60 colorectal cancer for the 2 study phases. Therefore, for the pilot phase, an additional 25 patients with acute myeloid leukemia and 55 patients with colorectal cancer will be enrolled in total across clinical sites. Mainz will attempt to recruit about two-third of the patients, that is, up to 20 patients with acute myeloid leukemia and up to 40 patients with colorectal cancer. IRST is expected to recruit 20 patients with colorectal cancer (1 site in Italy, IRST IRCCS) and 10 patients with acute myeloid leukemia. However, due to the COVID-19 health crisis, fewer patients are coming to the clinic and the number might have to be adjusted. Patients are enrolled for 3 months.

### Demographic, Medical, and Treatment Data

The clinical variables regarding medical and treatment data will be obtained from the patient’s medical records. The following data will be recorded for each patient at study entry through an electronic data capture system: (1) demographics include age, gender, weight, educational background, occupation, and family status; (2) medical and medication history include relevant medical and medication history and acute myeloid leukemia or colorectal cancer history including date of diagnosis, type, treatments, and disease status at study entry; (3) baseline conditions (comorbidities and symptoms) assessment; (4) history of oncological treatments and any residual toxicity relating to prior or current treatment; (5) performance status (Eastern Cooperative Oncology Group); and (6) physical examination.

### Instruments and Measures

#### Wearable Device and the Sensing Framework

The sensing infrastructure is an essential part of the ONCORELIEF ecosystem due to its role in providing and managing the data flow for all patients. The sensing framework describes the parameters that can be obtained from the wearable multisensor device as well as the ways to connect to them and encompasses four fundamental elements: (1) the whole set of smart sensors in the wearable device that includes sensors relevant both to the physiological parameters as well as to behavioral data (step count), (2) the patient’s smartphone ONCORELIEF mobile app (the patient-centered Guardian Angel app) (3) interim sensor data storage and further processing (AI algorithms for stress level based on heart rate variability parameters), and (4) ability of real-time (or almost real-time) data transmission from the wearable device via the Guardian Angel mobile app to the ONCORELIEF cloud.

The customizable smart wrist-worn sensor device developed by a partner of this project, MCS Datalabs, is equipped with diverse sensors. It is able to monitor vital data (optical heart rate = pulse rate and oxygen saturation by photoplethysmography and skin temperature) and motion data from a 3D accelerometer (step count as an output of the sensor). The wrist-worn device is configured and customized to meet the defined data and security requirements.

#### Psychosocial Instruments

Before the selection of the instruments for measuring patient needs, we selected the key needs that should be assessed for each type of cancer. The aim of this selection was to reduce the number of needs as much as possible to diminish the patients’ burden and make it possible to assess these needs through the Guardian Angel app and from the wearable. We started by identifying all needs documented in the scientific literature for acute myeloid leukemia and colorectal cancer, which resulted in a first list of needs. Next, the ONCORELIEF clinical partners (IRST and Mainz) filtered the needs based on the following criteria: (1) is it a common need (ie, affecting >10% of patients with the corresponding condition)? (2) can patients or caregivers self-report this need? (3) does the need have “intensity levels” or “grades” (1-5, or 1-10, or so on) or is it just “binary” (yes or no)? (4) is there more than one potential intervention available for this need? (5) could potential interventions bring noticeable improvement within the period of the pilot? (6) could this need be related to urgent care with action that needs to be taken in a very short timeframe? (7) is there a high risk from using AI (or training AI) for this need? (8) is this need a “must-have” patient need? From the app of these criteria resulted a final list of key needs presented in the first column of Table 1. Some needs are common to both cancers (anxiety, depression, fatigue, and subjective well-being) and others are specific, as shown in Table 1. Each patient will be assessed every 2 weeks over the 3-month participation in the ONCORELIEF study.

**Table 1:** Patient key needs and questionnaires.

Patient needs	Questionnaire	Description
Anxiety	Hospital Anxiety and Depression Scale (HADS) [12-14]	HADS is a 14-item measure designed to assess anxiety and depression. This study focuses only on the 7 items of anxiety (HADS-A). Each item is endorsed on a Likert-type scale (0-3), and the score obtained ranges from 0 to 21.
Depression	Patient Health Questionnaire-9 (PHQ-9) [12-14]	The PHQ-9 is the 9-item depression module from the full PHQ. The PHQ-9 score can range from 0 to 27, since each of the 9 items can be scored from 0 (not at all) to 3 (nearly every day).
Fatigue	Brief Fatigue Inventory (BFI) [15-17]	BFI is a 9-item measure to assess the subjective report of cancer-related fatigue severity and the impact of fatigue on daily functioning. BFI uses an 11-point scale (0=“no fatigue” to 10=“fatigue as bad as you can imagine”) to measure each specific symptom.
Appetite loss	EORTC-QLQ C30^a^ [18]	Assessed by the appetite loss item from EORTC-QLQ C30. In the past week, have you lacked appetite? (from 1=not at all to 4=very much).
Subjective well-being	World Health Organization Well-Being Index (WHO-5) [19]	WHO-5 is a generic global rating scale measuring subjective well-being. Each of the 5 items is scored from 5 (all of the time) to 0 (none of the time). The raw score ranges from 0 (absence of well-being) to 25 (maximal well-being).
Lack of sexual interest (only acute myeloid leukemia)	EORTC-SHQ-C22^b^ [20]	The EORTC SHQ-C22 is a 22-item scale designed to assess the physical, psychological, and social aspects of sexual health in patients with cancer and survivors of cancer. Each item is scored a 4-point Likert scale (not at all to very much).
Sleep problems (only acute myeloid leukemia)	Insomnia Severity Index (ISI) [21-23]	The ISI is composed of 7 items that evaluate sleep problems (the last item evaluates the level of distress caused by the sleep problem). Each of these items is rated on a 5-point Likert scale (not at all to extremely), and the time interval is “in the last 2 weeks.” Total scores range from 0 to 28, with high scores indicating greater insomnia severity.
Sore mouth (only acute myeloid leukemia)	EORTC-OH15^c^ [24]	The EORTC-OH15 assesses oral health quality of life in patients with any type of cancer. It has 15 items, containing one 8-item OH-QoL^d^ scale, 3 single items (sticky saliva or mouth soreness or sensitivity to food or drink), and two 2-item contingency scales regarding use (yes or no) and problems with dentures and reception of (yes or no) and satisfaction with information.
Colitis (only colorectal cancer)	EORTC-QLQ-CR29^e^ [25]	The European Organization for Research and Treatment of Cancer developed the colorectal QoL module as an adjunct to the generic EORTC QLQ-C30. This module consists of 4 scales and 19 individual items.
Hand-foot syndrome (only colorectal cancer)	EORTC-QLQ CIPN20^f^ [26]	The EORTC QLQ-CIPN20 is the common method for determining chemotherapy-induced peripheral neuropathy symptoms.
Neuropathy (only colorectal cancer)	EORTC-QLQ CIPN20	Same as previous scale.

^a^EORTC-QLQ C30: European Organization for Research and Treatment of Cancer Core Quality of Life questionnaire.

^b^EORTC-SHQ-C22: European Organization for the Research and Treatment of Cancer Sexual Health Questionnaire.

^c^EORTC-OH15: European Organization for the Research and Treatment of Cancer—Oral Health Questionnaire.

^d^QoL: quality of life.

^e^EORTC-QLQ-CR29: European Organization for Research and Treatment of Cancer—colorectal quality of life module.

^f^EORTC-QLQ CIPN20: European Organization for Research and Treatment of Cancer Quality of Life Questionnaire—Chemotherapy-Induced Peripheral Neuropathy 20-item scale.

The selection of the instruments for measuring the psychosocial needs described in Table 1 was based on three criteria: (1) sound reliability and construct validity, (2) reduced number of items, and (3) have already been translated and validated into Italian and German.

#### Measurement of the QoL and Well-being Index

The QoL and well-being composite index has a hierarchical structure of three levels: (1) global, (2) domains, and (3) needs. The first level represents the total QoL and well-being score. The second level includes 3 different need domains (emotional distress, psychophysiological, and physical). For example, the “emotional distress” domain includes the needs “depression” and “anxiety.” See Table 2 that presents the grouping of needs into domains and the instruments to assess the needs for each type of cancer, acute myeloid leukemia and colorectal cancer.

**Table 2:** Domains and key needs assessed for each type of cancer.

Domain	Acute myeloid leukemia needs	Colorectal cancer needs
Emotional distress	Depression Anxiety	Depression Anxiety
Psychophysiological	Sleep Fatigue Lack of sexual interest	Fatigue
Physical symptoms	Sore mouth Appetite loss	Colitis Appetite loss Peripheral neuropathy Hand-foot syndrome
Well-being	Well-being	Well-being

**Figure 4:**
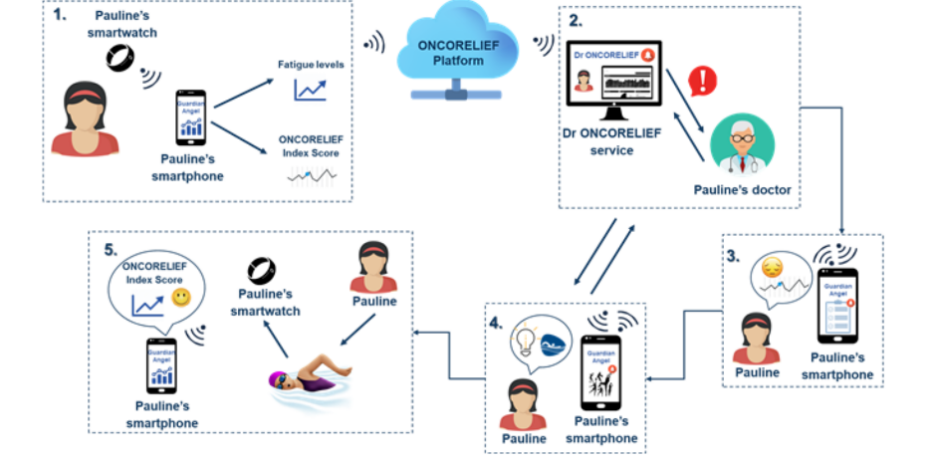
Pauline case illustration.

The different scores at each level will provide a set of metrics that will allow to identifying and monitoring QoL and well-being of patients with cancer. This multilevel approach will provide information about what domain or domains (eg, emotional distress) related with patient needs (eg, depression, anxiety) must be improved or to identify differences by gender, type of cancer, and country at the 3 levels. The global index results from averaging the scores of each domain, which are based on the scores obtained in each patient’s key needs. The QoL and well-being global score is converted on a 0-1000 scale and will allow a qualitative appreciation, which will range between 400 (very poor) and 900 or higher (very good). Clinical and psychosocial recommendations coming from the ONCORELIEF system are analyzed and decided by the health professional and are focused on specific needs rather than the overall score. However, it is expected that a positive change in specific unmet needs will be reflected positively on the QoL and well-being index global score. Below we describe a user scenario illustration based on a fictitious case (see Figure 4) as an example of how the ONCORELIEF digitally assisted workflow may hopefully work in a real-life setting.

#### User Scenario Illustration

Shortly after Pauline was diagnosed with stage 2 colorectal cancer, she had surgery followed by chemotherapy as adjuvant treatment. She accepted the opportunity to use the ONCORELIEF Guardian Angel app, although she didn’t particularly like the idea of entering much data into an app. Over time, the Guardian Angel app, having connected with her smart device, identified patterns, which had been correlated with the early onset of fatigue in patients like Pauline: she napped more than before and was less active. Additionally, her ONCORELIEF QoL and well-being global score had dropped from 700 to 500. Her HCP got notified of these patterns through the ONCORELIEF service and, after some consideration, sent a message to the patient. When Pauline got notified the next day, she read the accompanying text and realized from the color coding on her app that her QoL and well-being index score had dropped further, and upon further investigation, she read that it was 500 because of early fatigue and some mild depressive symptoms. It then occurred to her that this may have been the reason behind her low motivation and her reluctance to get up and go to work in the morning. She gave consent for her new information to be shared with her HCP and was provided with a few minutes’ worth of reading material, which made her feel somewhat better since she realized this is relatively common and not a huge cause of concern. Her HCP, after viewing the report on Pauline’s responses as well as her recent history via ONCORELIEF, gave a recommended intervention: gradual exercise increase and get support from psycho-oncology services. The HCP agreed and soon enough Pauline had the recommendation on her Guardian Angel app. She felt OK but not great—but then read that the exercise coach would motivate her based on her achievements but also that her exercises would be tailored to her unique needs and preferences. The accompanying content offered a variety of exercises: since she had not had an ostomy surgery and did not have a stoma bag, she could also swim in the local pool, and she liked that idea. Pauline started tracking her swimming sessions simply with a couple of taps at a time. The psycho-oncologist assessed Pauline’s depressive cognitions and beliefs and her lack of motivation and pleasure in doing daily activities and suggested some stress management skills to cope with depression. After a few weeks, both Pauline and her HCP could see her ONCORELIEF Index QoL and well-being score increasing slowly but steadily to 700.

### Ethics Approval

The protocol, informed consent, and any accompanying material provided to the patient were submitted to the ethical board of IRST (CEROM, Comitato Etico della Romagna, case number Prot. 8528/2021) and Mainz (Ethik-Kommission der Landesärztekammer Rheinland-Pfalz, case 2021-16122) for review. The pilot study was approved by both ethical boards. Any modifications made to the protocol, informed consent, or material provided to the patient after receipt of the Ethics Committee approval must also be submitted by the investigator to the committee in accordance with local procedures and regulatory requirements. It is the responsibility of the investigator to obtain written informed consent from each subject prior to entering the trial or, where relevant, prior to evaluating the subject’s suitability for the study.

The investigators will ensure that this study is conducted in full conformity with the current revision of the Declaration of Helsinki (last amended 64th WMA General Assembly, Fortaleza, Brazil, October 2013), with relevant regulations and with the ICH Guidelines for Good Clinical Practice E6 (R2), and other relevant local legislation.

## Results

The ONCORELIEF project started in January 2020 and will end in June 2023. The pilot study started on September 1, 2022. As of January 2023, we enrolled 33 patients with colorectal cancer and 7 patients with acute myeloid leukemia. As of January 2023, we have not yet started the data analysis. We expect to get all data in June 2023 and expect the results to be published in the second semester of 2023.

## Discussion

### Principal Findings

The main objective of ONCORELIEF is the development of a user-centered AI system to facilitate the input and integration of patient-related biopsychosocial data to improve posttreatment QoL, well-being, and health outcomes. We hope that this project will be feasible and will provide patients and health professionals with a valuable tool to improve the holistic rehabilitation of patients with cancer. There has been increasing use of eHealth tools in health care in general [5] and in patients with cancer in particular [4,27,28], but the main innovation of ONCORELIEF is that all data of patients with cancer will run on a digitally assisted workflow of posttreatment supportive patient care supported by an AI system. The core concept of the ONCORELIEF proposal is based on the collection of biopsychosocial patient data to provide patient-centered care and better support services to patients with cancer and survivors of cancer. ONCORELIEF will enable identifying, collecting, harmonizing, modeling, and eventually processing and analyzing data from diverse channels and domains, using Big Data and AI techniques to develop services and features that will be offered to all cancer stakeholders, with the aim to serve them with a continuous monitoring and assistance app, toward increasing the awareness of their QoL and well-being status. The overall output of ONCORELIEF has the potential to provide insights for HCPs and caregivers, as well as for patients, and to deliver recommendations toward more effective psychosocial support. Patients may be more motivated to improve their health and hasten their way back to normal, through the adoption of healthy lifestyles and stress management skills. The power of ONCORELIEF lies on a holistic model of health, capturing ongoing data from diverse sources (eg, patient-reported outcomes through a smartphone app, physiological signals from a wearable smart device, and demographic and medical data from health records) involving the biological, psychological, and social dimensions of the patient with the potential to offer the patient just-in-time recommendations to cope with the unmet needs and challenges of the cancer journey.

### Strengths

The strengths of the study are as follows: the platform and patient-assessment profile are continuously updated and is available 24/7, the study has the potential of less demand of medical consultations since ONCORELIEF gives support to the patient all the time, and the study contributes to better clinical outcomes.

### Limitations

The limitations of this study are resistance from some patients who are not familiar with digital devices, eHealth tools and procedures are not yet integrated into hospitals’ data evaluation and recording systems, and platforms and systems such as ONCORELIEF may emerge as extra tools that can be felt as complications in the day-to-day life of HCPs.

## Appendix

Multimedia Appendix 1 Project Evaluation (Peer-Review by European Commission).

## Abbreviations

AI: artificial intelligence
HCP: health care professional
IRST: Istituto Romagnolo per lo Studio dei Tumori “Dino Amadori”
QoL: quality of life

## Data Availability

Data sharing is not applicable to this paper as no data sets were generated or analyzed during this study.
