# Supplementary material for: Digital Guardian Angel Supported by an Artificial Intelligence System to Improve Quality of Life, Well-being, and Health Outcomes of Patients With Cancer (ONCORELIEF): Protocol for a Single Arm Prospective Multicenter Pilot Study
Source: JMIR Res Protoc. 2023 Apr 21;12:e45475. doi: 10.2196/45475 (PMC10163393; doi:10.2196/45475)
Supplement: Multimedia Appendix 1 [file resprot_v12i1e45475_app1.pdf]

Associated with document Ref. Ares(2019)4762874 - 22/07/2019

Proposal Evaluation Form

EUROPEAN COMMISSION

Horizon 2020 - Research and Innovation Framework Programme

Evaluation Summary Report - Research and innovation actions/Innovation actions

**Call:**	H2020-SC1-DTH-2019
**Type of action:**	RIA
**Proposal number:**	875392
**Proposal acronym:**	ONCORELIEF
**Duration (months):**	36
**Proposal title:**	A digital guardian angel enhancing cancer patient's wellbeing and health status improvement following treatment.
**Activity:**	SC1-DTH-01-2019

N.	Proposer name	Country	Total Cost	%	Grant Requested	%
1	EXUS SOFTWARE LTD	UK	701,250	14.39%	701,250	14.39%
2	MAGGIOLI SPA	IT	409,375	8.40%	409,375	8.40%
3	INNOSYSTEMS M.IKE.	EL	368,750	7.57%	368,750	7.57%
4	CARE ACROSS LTD	UK	418,750	8.59%	418,750	8.59%
5	ETHNIKO KENTRO EREVNAS KAI TECHNOLOGIKIS ANAPTYXIS	EL	256,250	5.26%	256,250	5.26%
6	SUITE5 DATA INTELLIGENCE SOLUTIONS LIMITED	CY	406,875	8.35%	406,875	8.35%
7	FRAUNHOFER GESELLSCHAFT ZUR FOERDERUNG DER ANGEWANDTEN FORSCHUNG E.V.	DE	461,250	9.47%	461,250	9.47%
8	UNIVERSITAETSMEDIZIN DER JOHANNES GUTENBERG-UNIVERSITAET MAINZ	DE	378,375	7.77%	378,375	7.77%
9	MCS DATALABS	DE	615,750	12.64%	615,750	12.64%
10	FCIENCIAS.ID - ASSOCIACAO PARA A INVESTIGACAO E DESENVOLVIMENTO DE CIENCIAS	PT	221,875	4.55%	221,875	4.55%
11	TIME.LEX	BE	205,000	4.21%	205,000	4.21%
12	ALMA MATER STUDIORUM - UNIVERSITA DI BOLOGNA	IT	181,250	3.72%	181,250	3.72%
13	ISTITUTO SCIENTIFICO ROMAGNOLO PERLO STUDIO E LA CURA DEI TUMORI SRL	IT	247,500	5.08%	247,500	5.08%
	Total:		4,872,250		4,872,250	

Abstract:
The burden of cancer is rising globally and is estimated to have reached 18.1 million new cases and 9.6 million cancer deaths in 2018. Despite the rising cancer incidence, improvements in early detection and therapeutic treatment have improved cancer survival. As a consequence, the number of cancer survivors is increasing globally, creating the need to improve not only treatment but also wellness and follow-up care. Cancer treatment often involves combined modalities such as surgery, chemotherapy, and radiotherapy. In the past decades, more effective and targeted therapeutic modalities and less destructive cancer treatments have been developed such as immunotherapy and drug-targeted therapy. Even so, cancer and its treatment have important physical and psychosocial sequelae. ONCORELIEF is a 36-month action that will leverage the above 6 drivers in order to skillfully and methodologically overcome technical challenges, by introducing new approaches that will allow the utilization of big datasets in order to develop a user-centered AI System to facilitate the integration of QoL assessment instruments through the use of PROMs and PREMs in order to improve post-treatment health status, increase the wellbeing, and follow-up care of cancer patients. This will be achieved through an intuitive smart digital assistant (Guardian Angel), able to provide personalized support in post-treatment activities and tasks, suggest actions regarding the patients' overall health-status, improved wellbeing and active health-care and ultimately maintain him/her engaged on a wellness journey that will safeguard his/her health over the foreseeable prolonged post-cancer treatment period. To achieve this, ONCORELIEF builds on the combined knowhow of its interdisciplinary industry-driven consortium that brings together state-of-the-art technological skills, design thinking methodology and occupational psychology/health sciences.

Evaluation Summary Report

Evaluation Result

Total score: 14.50 (Threshold: 12)

Form information

SCORING

Scores must be in the range 0-5.

Interpretation of the score:

__0__ The __proposal fails to address the criterion__ or cannot be assessed due to missing or incomplete information.

__1 Poor.__ The criterion is inadequately addressed, or there are serious inherent weaknesses.

__2 Fair.__ The proposal broadly addresses the criterion, but there are significant weaknesses.

__3 Good.__ The proposal addresses the criterion well, but a number of shortcomings are present.

Associated with document Ref. Ares(2019)4762874 - 22/07/2019

***4 Very good.** The proposal addresses the criterion very well, but a small number of shortcomings are present.*

***5 Excellent.** The proposal successfully addresses all relevant aspects of the criterion.Any shortcomings are minor.*

Criterion 1 - Excellence

Score: **5.00** (Threshold: 4/5.00 , Weight: -)

The following aspects will be taken into account, to the extent that the proposed work corresponds to the topic description in the work programme:
Clarity and pertinence of the objectives
Soundness of the concept, and credibility of the proposed methodology
Extent that proposed work is beyond the state of the art, and demonstrates innovation potential (e.g. ground-breaking objectives, novel concepts and approaches, new products, services or business and organisational models)
Appropriate consideration of interdisciplinary approaches and, where relevant, use of stakeholder knowledge and gender dimension in research and innovation content

The objectives are clearly described, measurable and are pertinent to the objectives of the call topic.

Concept is presented very comprehensively, discussing precisely and highly convincing different aspects and components of the expected framework, and it is sound. Particular attention is paid to user-centred approach, holistic post-treatment, and personalized well-being through a human-like coaching experience, which is excellent.

The proposal adopts the design thinking methodology based on agile/iterative approach, which is convincingly explained and it is highly credible. Additionally, planned pilots' validation methodology is also credible and convincingly incorporated in the proposal. How PROMs is addressed is consistent with the envisaged key patient benefits, and the involvement of a multi-disciplinary team and healthcare professionals is very appropriate. The methodology also appropriately addresses the management of research data by securing a broad data collection from a wide range of reliable sources. Approaches towards security and privacy issues are very well addressed, confirming the adoption of a privacy-by-design approach, so that no sensitive data will leave device (or the side) of individuals, while all other data that will be transferred would require the user's consent and will be fully anonymized.

The analysis of the state of the art is comprehensive. The progress beyond the state of the art is well justified in the areas of Convolutional (CNN) and Recurrent Neural Network (RNN) technologies, which are relevant to the purpose of managing, modelling, processing and exploiting big data for the registration and analysis of MRI images to effectively monitor health status of individual patients, provide overall actionable insights at the point of care and improve quality of life after the cancer treatment. The description of several new products and methods that will be developed (e.g. personalized mobile application and seamless analytics over cross-origin data) demonstrate high innovative potential.

The proposal ensures appropriately combined know-how and interdisciplinary approaches.

The use of stakeholder knowledge is very good, incorporating patients, caregivers, psychologists and clinicians in addition to ICT stakeholders' knowledge.

Gender aspects are appropriately considered.

Criterion 2 - Impact

Score: **5.00** (Threshold: 4/5.00 , Weight: -)

The following aspects will be taken into account:
The extent to which the outputs of the project would contribute to each of the expected impacts mentioned in the work programme under the relevant topic
Any substantial impacts not mentioned in the work programme, that would enhance innovation capacity, create new market opportunities, strengthen competitiveness and growth of companies, address issues related to climate change or the environment, or bring other important benefits for society
Quality of the proposed measures to:
- exploit and disseminate the project results (including management of IPR), and to manage research data where relevant
- communicate the project activities to different target audiences

The outputs of the project will contribute to each of the expected impacts mentioned in the work programme under the relevant topic in a highly effective manner. For instance, the commitment to achieve 100% privacy preservation at generic analytics layers due to anonymisation techniques used for the impact on "emerging data driven analytics and advanced simulation methods to study causal mechanisms and improve forecasts of ill-health, identification of disease trajectories and relapse" is commendable. Additionally, the envisaged 20% reduction in the occurrence of health disorders and co-morbidities due to improved monitoring of individuals related with the impact of "preventative strategies to have a real effect of reducing the occurrence of health disorders and co-morbidities associated with cancer treatment" is particularly important. Appropriate impact measurable indicators are proposed.

A good range of different relevant impacts and benefits (Societal Benefits, Impact on Health Laws and Policies, Economic, Business and Market Impact, National, European and International Impact and contributions to Standardisation and Research and Innovation) are identified. Strong impact on Health Laws and Policies is fully and comprehensively justified from the point of view of several important and challenging areas.

Overall exploitation objectives and activities are very well identified and are highly effective. Additionally, individual exploitation plans are adequately presented.

The proposal presented well-defined and highly effective dissemination and multi-channel communication strategies targeted to wide multi-stakeholder audiences. Measurable results are attributed to the core communication and dissemination activities, that are very well suited to secure how stakeholder groups are targeted and dissemination channels are utilised. While the dissemination plan provides much detail, it

Associated with document Ref. Ares(2019)4762874 - 22/07/2019

does not specifically align to the scientific field of cancers that have been chosen.

The management of IPR is well addressed.

Criterion 3 - Quality and efficiency of the implementation

Score: **4.50** (Threshold: 3/5.00 , Weight: -)

The following aspects will be taken into account:
Quality and effectiveness of the work plan, including extent to which the resources assigned to work packages are in line with their objectives and deliverables
Appropriateness of the management structures and procedures, including risk and innovation management
Complementarity of the participants and extent to which the consortium as a whole brings together the necessary expertise
Appropriateness of the allocation of tasks, ensuring that all participants have a valid role and adequate resources in the project to fulfil that role

The work plan is highly effective and very appropriate. Work package descriptions are well presented and the resources assigned are fully in line with the objectives and the committed deliverables. Project milestones with means of verification are well suited for monitoring project advances and the achievement of the objectives.

Project management approach, structures and procedures are convincing and appropriate, including effective quality assurance and control procedures.

Risk Management is convincingly presented. The risks will be constantly assessed and evaluated within the whole project duration. Identified risks and their related mitigation measures are appropriate. However, the risk that the AI will not provide the required level of output quality is not sufficiently addressed.

Whilst an Innovation Manager is appointed, the proposal provides insufficient information on the Innovation Management approach and procedures.

The consortium members cover all the required research areas in a well-balanced way involving representatives from industry, academia, research institutions, and end-users. The necessary expertise of consortium members is highly relevant and fully in line with the proposed activities and the expected results.

The allocation of tasks is appropriate to ensure that all participants have a valid role and adequate resources in the project to fulfil that role.

Scope of the proposal

Status: **Yes**

Comments (in case the proposal is out of scope)

Not provided

Operational Capacity

Status: **Operational Capacity: Yes**

If No, please list the concerned partner(s), the reasons for the rejection, and the requested amount.

Not provided

Exceptional funding of third country participants/international organisations

A third country participant/international organisation not listed in General Annex A to the Main Work Programme may exceptionally receive funding if their participation is essential for carrying out the project (for instance due to outstanding expertise, access to unique know-how, access to research infrastructure, access to particular geographical environments, possibility to involve key partners in emerging markets, access to data, etc.). (For more information, see the Online Manual)

Based on the information provided in the proposal, we consider that the following participant(s)/international organisation(s) that requested funding should exceptionally be funded:
(Please list the Name and acronym of the applicant, Reasons for exceptional funding and the Requested grant amount.)

Not provided

Based on the information provided in the proposal, we consider that the following participant(s)/international organisation(s) that requested funding should NOT be funded:
(Please list the Name and acronym of the applicant, Reasons for exceptional funding and the Requested grant amount.)

Not provided

Use of human embryonic stem cells (hESC)

Status: **No**

If yes, please state whether the use of hESC is, or is not, in your opinion, necessary to achieve the scientific objectives of the proposal and the reasons why. Alternatively, please state if it cannot be assessed whether the use of hESC is necessary or not because of a lack of information.

Not provided

Overall comments

The proposal is above threshold in all criteria and overall.

European Commission

FUNDING & TENDERS PORTAL DIGITAL SEAL

Digitally sealed by the European Commission
Date: 2019.07.22 17:02:12 CEST

This document is digitally sealed. The digital sealing mechanism uniquely binds the document to the modules of the Funding & Tenders Portal of the European Commission, to the transaction for which it was generated and ensures its integrity and authenticity.

Any attempt to modify the content will lead to a breach of the electronic seal, which can be verified at any time by clicking on the digital seal validation symbol.
